# Immediate and Delayed Cochlear Neuropathy after Noise Exposure in Pubescent Mice

**DOI:** 10.1371/journal.pone.0125160

**Published:** 2015-05-08

**Authors:** Jane Bjerg Jensen, Andrew C. Lysaght, M. Charles Liberman, Klaus Qvortrup, Konstantina M. Stankovic

**Affiliations:** 1 Eaton-Peabody Laboratories and Department of Otolaryngology, Massachusetts Eye and Ear Infirmary, Boston, MA, 02114, United States of America; 2 Department of Otology and Laryngology, Harvard Medical School, Boston, MA, 02115, United States of America; 3 Program in Speech and Hearing Bioscience and Technology, Division of Health Science and Technology, Harvard and Massachusetts Institute of Technology, Boston, MA, 02139, United States of America; 4 Department of Biomedical Sciences, CFIM, University of Copenhagen, 2200, Copenhagen N, Denmark; University of California, Irvine, UNITED STATES

## Abstract

Moderate acoustic overexposure in adult rodents is known to cause acute loss of synapses on sensory inner hair cells (IHCs) and delayed degeneration of the auditory nerve, despite the completely reversible temporary threshold shift (TTS) and morphologically intact hair cells. Our objective was to determine whether a cochlear synaptopathy followed by neuropathy occurs after noise exposure in pubescence, and to define neuropathic versus non-neuropathic noise levels for pubescent mice. While exposing 6 week old CBA/CaJ mice to 8-16 kHz bandpass noise for 2 hrs, we defined 97 dB sound pressure level (SPL) as the threshold for this particular type of neuropathic exposure associated with TTS, and 94 dB SPL as the highest non-neuropathic noise level associated with TTS. Exposure to 100 dB SPL caused permanent threshold shift although exposure of 16 week old mice to the same noise is reported to cause only TTS. Amplitude of wave I of the auditory brainstem response, which reflects the summed activity of the cochlear nerve, was complemented by synaptic ribbon counts in IHCs using confocal microscopy, and by stereological counts of peripheral axons and cell bodies of the cochlear nerve from 24 hours to 16 months post exposure. Mice exposed to neuropathic noise demonstrated immediate cochlear synaptopathy by 24 hours post exposure, and delayed neurodegeneration characterized by axonal retraction at 8 months, and spiral ganglion cell loss at 8-16 months post exposure. Although the damage was initially limited to the cochlear base, it progressed to also involve the cochlear apex by 8 months post exposure. Our data demonstrate a fine line between neuropathic and non-neuropathic noise levels associated with TTS in the pubescent cochlea.

## Introduction

Noise-induced hearing loss is a growing epidemic worldwide, with over 1.6 million new cases yearly [1]. Recent research in mice and guinea pigs has shown that even exposure to moderate noise levels, previously thought to cause only temporary hearing loss, can result in immediate and irreversible loss of cochlear neurons [2,3].

High noise levels cause permanent threshold shift (PTS), as assessed using behavioral threshold audiometry or physiologic metrics in both animals and humans. The common physiological metrics are auditory brainstem evoked responses (ABRs) and otoacoustic emissions (OAEs). ABR is an auditory-evoked, surface potential consisting of several waves, the first of which reflects the summed activity of the cochlear nerve. OAEs are generated by outer hair cells and serve as a measure of their integrity. PTS is seen in permanent elevation of both ABR and OAE thresholds, with multiple structural correlates within the cochlea: loss of hair cells or their stereocilia, collapse of the organ of Corti, loss of cochlear neurons from the spiral ganglion, loss of fibrocytes within the spiral ligament, and atrophy of the stria vascularis [4–6].

Until recently, moderate noise levels that cause temporary threshold shift (TTS)—defined as a temporary elevation in physiologic or behavioral thresholds that recovers to pre-exposure levels within 1 to 2 weeks post exposure—were thought to cause only temporary structural changes, such as reversible collapse of outer hair cells (OHCs), and the supporting cells of the organ of Corti [4–6]. However, recent work in adult rodents has shown that noise-induced TTS can lead to permanent auditory neuropathy characterized by irreversible reduction of ABR wave I amplitude, and immediate loss of afferent synapses on inner hair cells (IHCs), followed by a delayed loss of the associated cell bodies of cochlear neurons [2]. This noise-induced cochlear neuropathy is considered primary because hair cells and other cochlear cells survive and appear normal. This neuropathy is also known as “hidden” hearing loss [7], as it preferentially affects high-threshold neurons [8] that do not affect cochlear thresholds, yet are likely essential for hearing in noisy backgrounds.

Since primary cochlear neuropathy has thus far been carefully studied only after noise exposure in young adulthood, we explored this phenomenon after noise exposure in pubescence [9], because the peri-pubescent cochlea is known to have enhanced sensitivity to PTS in various animal models [10–16], and hearing loss is predicted to rise in juveniles [17]. We defined neuropathic and non-neuropathic noise levels associated with TTS in pubescent CBA/CaJ mice, and characterized cochlear physiologic and histologic changes from 24 hours to 16 months post exposure. Neuropathic noise levels were separated by only 3 dB from non-neuropathic levels. The damaging effects of neuropathic noise targeting the cochlear base were initially limited to the base, but with time progressed to also affect the cochlear apex. Our findings may have implications for regulating noise levels to prevent “hidden” hearing loss associated with TTS, and for future omics-based studies to decipher the underlying molecular mechanisms.

## Methods

### Animal groups and noise exposures

We used CBA/CaJ mice because they have good cochlear sensitivity through most of their life [18]. Six week old males (purchased from Jackson Laboratories) were exposed for 2 hours to an octave-band noise (8–16 kHz) at various sound levels from 94 to 100 dB SPL, to identify the highest sound pressure level (SPL) that causes TTS without a permanent decrease in ABR wave I amplitude (i.e. “non-neuropathic” noise) and the lowest SPL that causes TTS and a permanent decrease in ABR wave I amplitude (i.e. “neuropathic” noise). Animals were awake during exposures and held unrestrained in small cages. Sound exposure was created by a white-noise source, filtered, amplified and delivered through a horn attached to the top of the exposure booth. Exposure levels were measured in each cage with a 0.25 inch Brüel and Kjær condenser microphone.

A total of 142 animals were used to study short and long-term effects of these noise levels on cochlear physiology and histology compared to age-, gender- and strain-matched unexposed control mice. All procedures were approved by the Institutional Animal Care and Use Committee at the Massachusetts Eye and Ear Infirmary.

### Cochlear physiology: ABR and DPOAE

Auditory function was evaluated in unexposed and noise-exposed animals using ABR and DPOAEs. Animals were tested at 6 hours, 2 weeks, 4 weeks, 10 months and 16 months post exposure. Five to nine mice were tested at each time point for each noise exposure group. Mice were anesthetized with ketamine (100 mg/kg) and xylazine (10 mg/kg) intraperitoneally. DPOAEs were measured as ear canal pressure in response to two tones presented into the ear canal (f1 and f2, with f2/f1 = 1.2) at half octave steps, from f2 = 5.66–45.25 kHz, and in 5 dB intensity increments from 15 to 80 dB SPL. ABR responses were measured between subdermal electrodes, after amplification (10,000X) and filtering (0.3–3.0 kHz): positive behind the ipsilateral pinna, negative at the vertex and ground at the tail. For each frequency and sound level, 512 responses were recorded and averaged using custom LabVIEW data-acquisition software run on a PXI chassis (National Instruments Corp., Austin, Texas). By visual inspection of stacked waveforms, threshold was defined from lowest to highest SPL, as the first level at which a repeatable wave I was detected. ABR wave I amplitude (measured peak-to-peak) was determined for 80 dB SPL exposures to 11.3 kHz and 32.0 kHz frequency using the ABR Peak Analysis software (Bradley Buran, Eaton-Peabody Laboratories).

### Histological processing and analysis

Deeply anesthetized animals were perfused intracardially with either 4% paraformaldehyde (PFA) for cochlear whole mounts, or with 2.5% glutaraldehyde (GLUT)/1.5% PFA in 0.1M phosphate buffer (PB) (pH = 7.3) for resin embedding. Both bullae were opened, and the round and oval window membranes punctured to allow gentle intracochlear perfusion with the same fixative as used intracardially. Cochleae were extracted and post-fixed for 2 hours in 4% PFA for whole mounts, or in 2.5% GLUT/1.5% PFA overnight for resin embedding. Cochleae were decalcified in 0.12M EDTA or in 0.12M EDTA with 0.1 M PB and 1% GLUT (pH = 7), respectively, for 3 days at room temperature.

#### Cochlear whole mounts and quantitative confocal immunohistochemistry

The spiraling organ of Corti was microdissected into six pieces and immunostained with primary antibodies targeting C-terminal binding protein expressed in presynaptic ribbons and nuclei of hair cells (mouse IgG1 anti-CtBP2 at 1:200, BD Transduction Labs. #612044) [19], followed by double labeling with secondary antibodies AlexaFlour 568 rabbit anti-mouse coupled with AlexaFlour 568 donkey anti-rabbit. Whole mount samples were first imaged using low-power light microscopy to allow location-to-frequency mapping, using a custom plug-in to ImageJ. Guided by the cochlear frequency map, confocal microscopy (Leica SP5) was performed in the 11.3 kHz and 32.0 kHz regions while focusing on the presynaptic ribbons in the basolateral portion of IHCs; a glycerol-immersion 63x objective at 3.17 digital zoom and a 0.25 μm z-step were used. For each frequency region, in each cochlea, z-stacks were acquired at 3 adjacent areas, each containing ~10 IHCs in a row. Z-stacks were transferred to the Amira imaging software (Visage Imaging, version 5.2.2). The connected components and iso-surface functions were used to quantify ribbon numbers, which were expressed as a mean (ribbons/IHC) of the 3 areas per frequency region.

#### Cochlear osmication, resin embedding and stereology based counts

Decalcified cochleae were post-fixed in 1% osmium tetroxide in distilled water (dH_2_O) for 1 hour, then washed in dH_2_O and sequentially dehydrated with 70%, 95% and 100% ethanol, incubated in propylene oxide for 30 min, infiltrated with increasing concentrations of araldite, oriented in molds and embedded in 100% araldite. After hardening in a 60°C oven for at least 2 days, cochleae were cut in 20 μm-thick serial sections in a plane parallel to the midmodiolar axis using a Leica RM 2155 microtome. Sections were mounted in permount on glass slides and coverslipped.

The density of peripheral axons and cell bodies of spiral ganglion neurons (SGNs) in the cochlear apex (~11 kHz) and base (~32 kHz) was estimated using principles of unbiased stereological techniques. Axons were quantified based on the 2D fractionator technique [20], and SGNs were quantified based on the optical fractionator technique [21]. Stereological analyses were performed on live video images, obtained through a 100x oil immersion objective (NA = 1.35), projected to a computer monitor. The image was merged with the Computer Assisted Stereological Toolbox (C.A.S.T.-GRID software, The International Stereology Center at Olympus, Denmark), and a motorized stage moved sections in well-defined steps in the *x* and *y* axes. Before every count, the area of interest was delineated at low magnification, by drawing a line around all cells of interest. The C.A.S.T.-GRID software would randomly assign unbiased counting frames in a systematic, random raster pattern covering the entire area of interest, i.e. the first counting frame was placed randomly and the following were subsequently placed systematically, separated by the step distances, d*x* and d*y*, between frames. The area (a) of each counting frame was 177.3 μm^2^ and the total number of frames (n) was noted for all sessions.

Quantification of peripheral axons per area (**N**_**A**_) in the cochlear apex and base was performed on tangential sections through the osseous spiral lamina that contained peripheral axons but not SGN somata. Markers were placed on myelinated, peripheral axons, in the middle depth of a section (approximately 10 μm in depth), at the best focal plane within the counting frame. Since the counting frames constitute a well-known fraction of the cross-sectional area of interest, the total number of axons per mm^2^ (**N**_**A**_) is estimated as the number of axons counted (Q) divided by the area of the sampling fraction: **N**_**A**_ **= Q**/(a * n).

SGN quantification per volume (**N**_**V**_) was performed on every second slide on an average of eight slides in the upper apex (~11 kHz region) and twelve slides in the lower base (~32 kHz). Sampling was performed within the disector height (h), with the first disector focal plane defined as starting 3 μm below the section top and extending 15 μm down into the sample (h = 15 μm). All counts were performed within the height of the dissector, with markers placed on the nucleoli of SGNs. Nucleoli that came into focus between the top and bottom optical planes, or within the bottom plane, were included; nucleoli in focus within the top plane were excluded. After systematic sampling at all levels, the density of SGNs per mm^3^ (N_V_) is estimated as the total number of counted SGNs (Q) divided by the volume of the dissector: **N**_**V**_ = Q/(a * n * h).

The same experienced observer (J.B.J.) performed quantification of both peripheral axons and cell bodies of SGNs, and histopathological evaluation of cochlear IHCs and supporting cells. The observer was blinded to animal age and noise exposure.

### Statistical analysis

All histological and physiological data were analyzed for statistical significance using R free software, ver. 3.1.1. Group means for noise exposed and unexposed groups were compared using two-way ANOVA, and post hoc two-sample t-test with pooled-variance (or Wilcoxon rank-sum test when N < 7) with Benjamini-Hochberg (BH) multiple comparisons correction. Trends with time, within the same group, were calculated with one-way ANOVA and post hoc Dunnett’s multiple comparisons test, with all post hoc comparisons being made against the earliest time point. Results are expressed as mean ± standard deviations (SD). Differences between means were considered statistically significant when *p* < 0.05.

## Results

### Noise-induced Cochlear Dysfunction: inferring synaptopathic exposure

**Fig 1:**
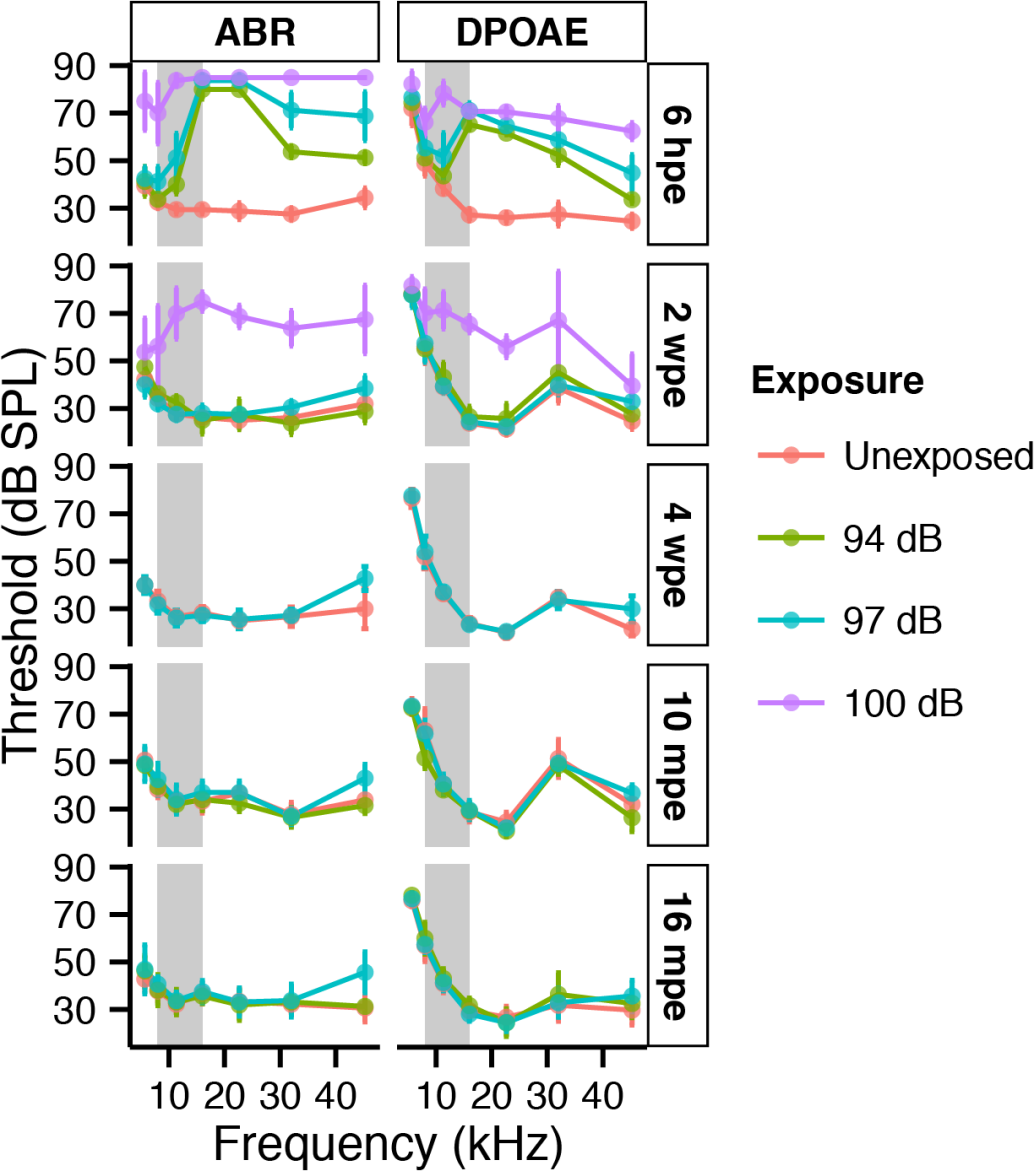
Cochlear thresholds are either permanently or temporarily shifted following neuropathic noise. ABR (left column) and DPOAE (right column) thresholds resulting from exposure of 6 week old mice to 8–16 kHz noise for 2 hours at 100 dB SPL (purple), 97 dB SPL (neuropathic exposure, blue) and 94 dB SPL (non-neuropathic exposure, green) compared to unexposed controls (red). Thresholds are shown as group means ± SD. The noise band is depicted as a gray vertical bar. Time post exposure (pe) is specified in hours (h), weeks (w) or months (m) in vertical boxes on the right side of each row. N = 5–9 mice per time point and noise exposure.

**Fig 2:**
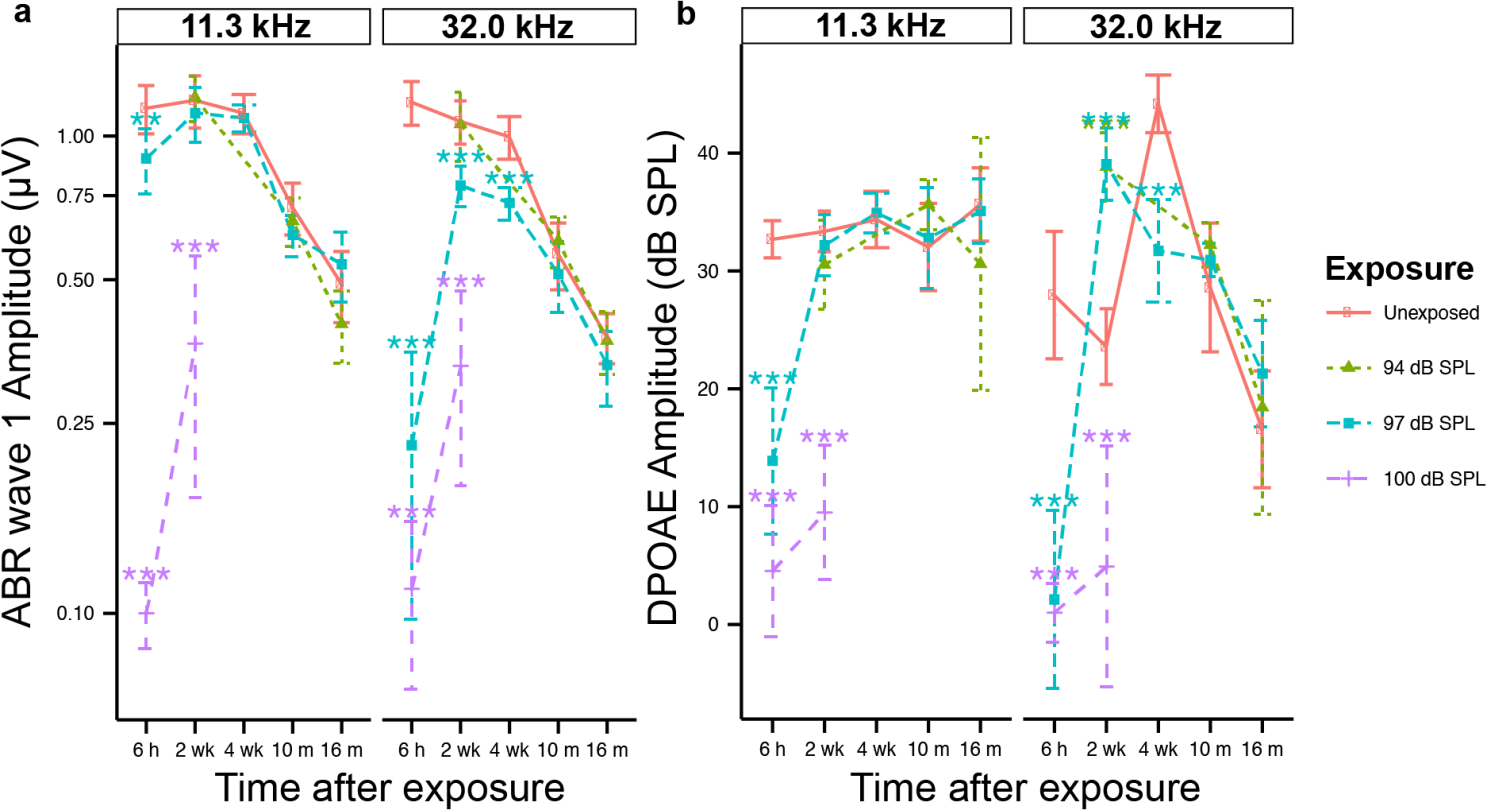
Neural response is reduced following neuropathic noise. ABR wave I peak-to-peak amplitudes (**a**) and DPOAE amplitudes (**b**) in response to 11.3 kHz tones (left column) and 32.0 kHz tones (right column) recorded from 6 hours to 16 months post exposure to noise causing PTS (100 dB SPL, purple), neuropathic noise causing TTS (97 dB SPL, blue), non-neuropathic noise causing TTS (94 dB SPL, green), and unexposed animals (red). ABR amplitudes are in response to 80 dB SPL tones (the maximum level tested) and DPOAE amplitudes are in response to 80 dB SPL tones. Noise parameters are as in Fig 1. Statistical significance of the group differences is indicated by asterisks: *: *p < 0*.*05*, **: *p < 0*.*01*, ***: *p < 0*.*001*. Data are group means ± SD. N = 5–9 mice per time point for noise-exposed and control groups.

We used a combination of sound-evoked responses from the auditory nerve and outer hair cells to infer cochlear synaptopathy in 6 week old pubescent mice (Figs 1 and 2). Prior work in noise-exposed 16 week old adult mice has shown that, despite return of cochlear thresholds to baseline, suprathreshold neural responses can remain suppressed long after exposure, and that these decrements in ABR wave 1 amplitude are indicative of permanent noise-induced loss of cochlear nerve terminals [2,22].

To make our study in pubescent mice comparable to that in 10 week older mice mice [2], we used the same duration (2 hours) and frequency band (8–16 kHz) of noise while varying noise levels. Two-way ANOVA of ABR and DPOAE thresholds returned highly significant exposure and time effects (*p < 0*.*001*). Importantly, 100 dB SPL noise that caused TTS in young adults aged 16 weeks [2] resulted in PTS in pubescent mice aged 6 weeks (Fig 1, purple line), with significant ABR threshold shifts of > 20 dB (*p < 0*.*001* by BH corrected pooled-variance t-test) at all but the lowest frequency tested. Exposure to 97 dB SPL noise induced mostly a TTS (Fig 1, blue line): ABR threshold shifts of 20–58 dB were highly significant (*p < 0*.*001*) above 10 kHz at 6 hours post exposure, but returned to normal 2 weeks post exposure at all frequencies except 45.2 kHz. An approximately 10 dB (*p < 0*.*001*) threshold shift at 45.2 kHz remained 4 weeks after exposure. Exposure to 94 dB SPL noise resulted in up to 55 dB TTS in ABR thresholds, with recovery across the frequency spectrum by 2 weeks post exposure (Fig 1, green line). DPOAE thresholds mirrored the ABR measurements in all 3 groups, though the shifts were slightly smaller, as reported previously [12]. DPOAE suprathreshold magnitudes were significantly (*p < 0*.*001*) reduced by the 100 dB SPL exposure (Fig 2B, purple line), however no permanent DPOAE response decrements resulted from the 97 and 94 dB exposures (Fig 2B, blue and green line), suggesting full recovery of outer hair cell function.

To gain insight into possible synaptic loss, we quantified changes in suprathreshold ABR wave I amplitude (Fig 2A) in two frequency regions—one (32.0 kHz) at the cochlear region of maximum neuropathy in adult ears, and the other (11.3 kHz) within the unaffected region in the apical half of the cochlea [2]. Two-way ANOVA identified highly significant (*p < 0*.*001*) exposure and time effects at both frequencies. For 94 dB SPL noise (Fig 2A, green line) there were no significant *changes* in wave I amplitude at any frequency, compared to age-matched controls (red line). In contrast, exposure to PTS-causing noise of 100 dB SPL (purple line) induced significant (*p < 0*.*001*) decreases in both frequency regions, when measured 2 wks post exposure. Effects of the 97 dB noise were intermediate. Wave I amplitudes at 32.0 kHz decreased by over 75% (*p < 0*.*001*) 6 hr after exposure, 22.5% + 10.2% (*p < 0*.*001*) at 2 weeks after exposure, and 20.1% + 10.2% (*p < 0*.*001*) at 4 weeks after exposure. Beyond 10 months post exposure, ABR wave 1 amplitude was similar in our control and noise exposed groups. This observation has to be viewed cautiously because our animal facility was under construction for several weeks during this period, thus “control” animals may have been exposed to neuropathic noise. Importantly, strong correlation has been described between ABR wave I amplitude and cochlear neuropathy in young adult mice purposely exposed to noise of the same duration and frequency [2]. ABR wave I amplitudes in the 11.3 kHz region were largely unaffected by the 97 dB SPL exposure, except at 6 hours post exposure when threshold elevations were also seen in the DPOAEs (*p < 0*.*01*) (Fig 2B, blue line). Of note, unexposed mice demonstrate progressive decrease in DPOAE amplitude with age (Fig 2B, red line) in the 32.0 kHz region. A similar age-related decline in DPOAE amplitude has been described in 22–26 months old CBA/CaJ mice [23] and 17 months old CBA/JNia mice [24].

### Cochlear synaptopathy: immediate and delayed effects

Basolateral regions of the IHC contain presynaptic ribbons that tether synaptic vesicles near the active zone, where voltage-gated calcium channels are clustered [25–28]. The ribbons regulate exocytotic release of glutamate-filled vesicles into the synaptic cleft, and are necessary for the temporal precision that characterizes sound-evoked SGN responses [19,29,30]. Malfunction or misplacement of ribbons decreases the sensitivity of the auditory nerve and desynchronizes their responses to stimulus onsets [19,31,32]. The peripheral axons of SGNs are unbranched, and, although each SGN synapses on only one IHC, each IHC is contacted by 10–30 SGNs, depending on frequency regions and species [25,33,34].

**Fig 3:**
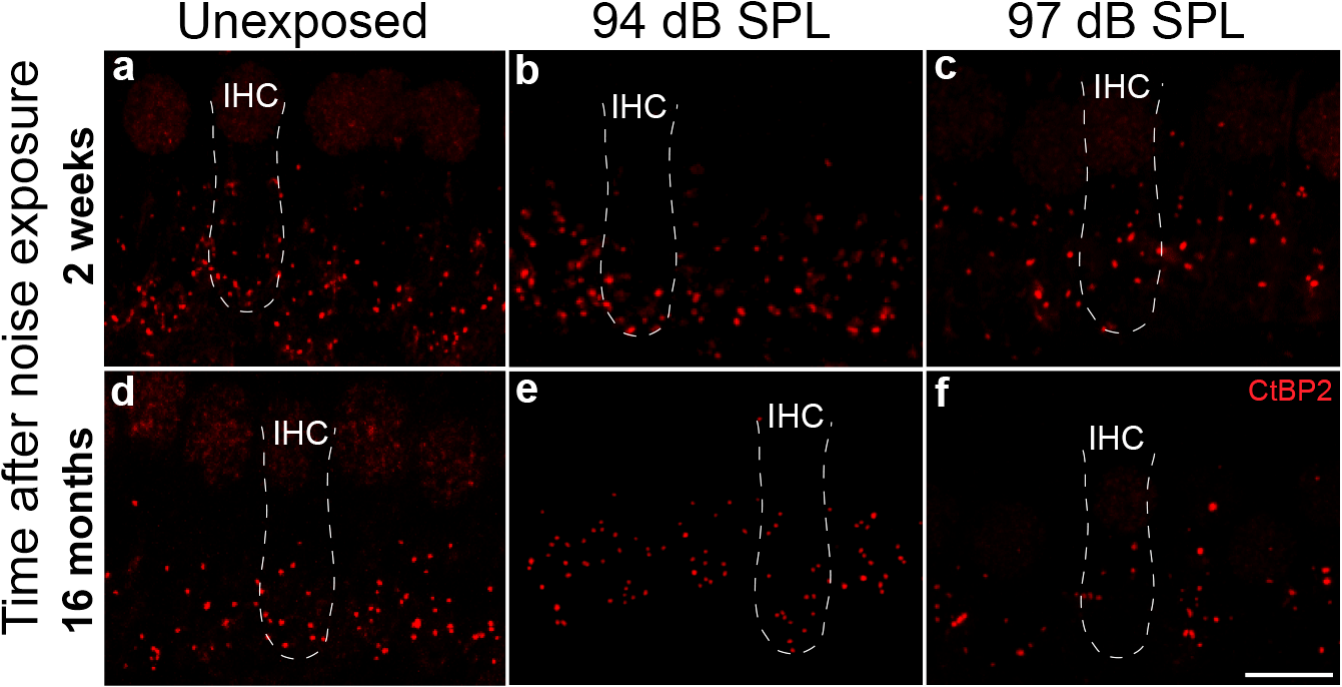
Confocal imaging shows a reduction in CtBP2 stained presynaptic ribbons following neuropathic noise. Short-term (**a**-**c**) and long-term (**d**-**f**) loss of presynaptic ribbons in inner hair cells (IHCs) after exposure to neuropathic (97 dB SPL) noise compared to age-matched animals exposed to non-neuropathic noise (94 dB SPL) and unexposed controls. Presynaptic ribbons are labeled with anti-CtBP2 antibodies (red). Confocal images are maximal projections of z-stacks of ribbons within 4–5 IHCs in the 32 kHz region. Dashed lines depict IHC outlines. Scale bar: 10 μm (**a**-**f**).

We evaluated synaptic ribbons in cochlear whole mount samples labeled with anti-CtBP2 antibody (Fig 3). In unexposed ears (Fig 3A and 3D) and in ears exposed to 94 dB SPL noise (Fig 3B and 3E), ribbons were found along the IHC basolateral membrane, at levels below the IHC nuclei. Exposure to 97 dB SPL noise caused a loss of ribbons at 2 weeks post exposure (Fig 3C), with additional losses at 16 months post exposure (Fig 3F). Ribbons tended to undergo a change in contour and be more displaced in the apical direction after neuropathic noise exposure than after non-neuropathic noise exposure or in control ears.

**Fig 4:**
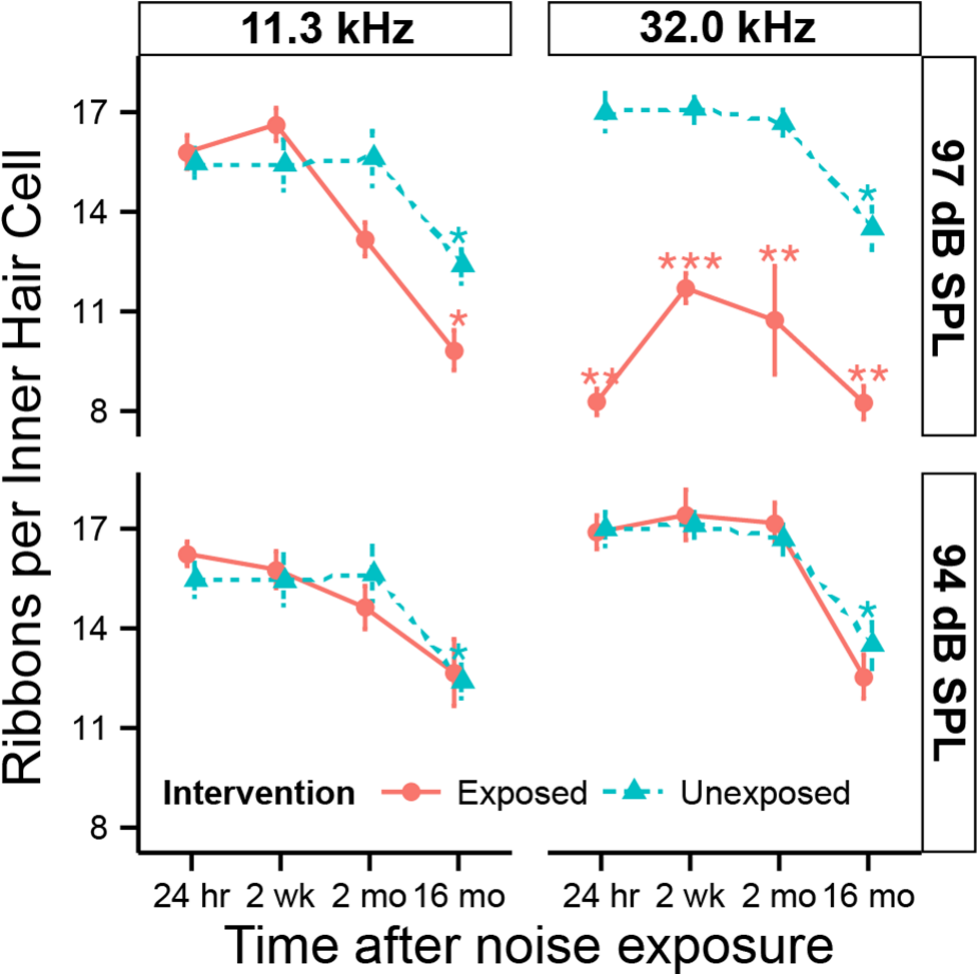
Numbers of presynaptic ribbons are reduced after neuropathic noise. Synaptic counts in inner hair cells in the 11.3 kHz (left) and 32.0 kHz (right) regions following exposure to neuropathic (97 dB SPL) or non-neuropathic (94 dB SPL) noise. Statistical significance of the group differences is indicated by asterisks: *: *p < 0*.*05*, **: *p < 0*.*01*, ***: *p < 0*.*001*. Data are group means ± SD. N = 6–7 animals per time and noise exposure.

Quantification of ribbons in the 11.3 and 32.0 kHz regions revealed significant differences after exposure to neuropathic (Fig 4, top row) vs. non-neuropathic noise (Fig 4, bottom row). Two-way ANOVAs suggest highly significant (*p* < *0*.*001*) time effects at both frequencies, a significant exposure effect (*p* < *0*.*001*) at 32.0 kHz, and a significant time-exposure interaction term (*p* < *0*.*05*) at 11.3 kHz. Mean ribbons per IHC in controls were 15.5 + 0.5 at 11.3 kHz and 17.0 + 0.6 at 32.0 kHz. Within 24 hours after 97 dB SPL noise, ribbon counts at 32.0 kHz were reduced by 51.3% + 4.4% compared to unexposed ears (*p < 0*.*01* by BH corrected Wilcoxon rank sum test). Two weeks later, the reduction (31.6% + 3.6%) was still significant (*p < 0*.*001*) and persisted (38.9% + 6.2%) at 16 months (*p < 0*.*01*). In the 11.3 kHz region, there was no immediate ribbon loss after exposure to 97 dB SPL noise. However, the mean counts decreased 15.7% + 5.9% (*p > 0*.*05)* by 2 months after exposure, and the difference from control (20.7% + 6.3%) became significant (*p < 0*.*05*) by 16 months post exposure. For both frequency regions, there was no significant difference between ears exposed to 94 dB SPL and unexposed ears across all post-exposure times.

Irrespective of noise exposure, there was a gradual decline in ribbon numbers with age, as reported previously [22]. Ribbon counts in unexposed 17.5 month old animals (16 months “post exposure”) were reduced by 19.8% + 4.5% in the 11.3 kHz (*p < 0*.*01*) and by 20.5% + 5.1% in the 32.0 kHz region (*p < 0*.*001*) compared to 6 week old mice (24 hours “post exposure”).

**Fig 5:**
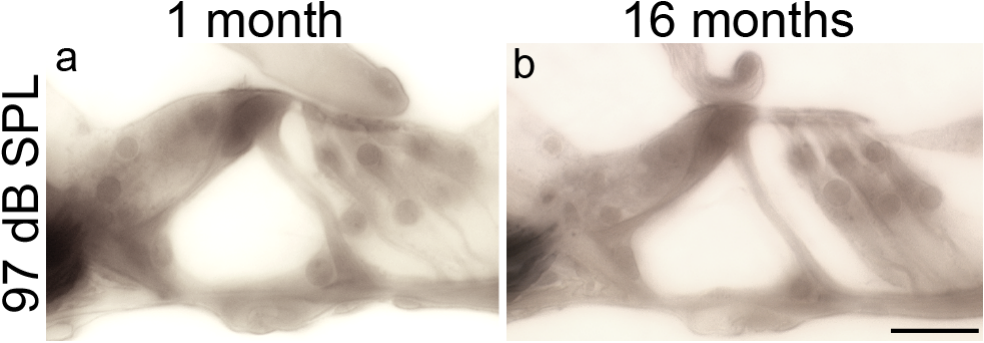
Cochlear sensory cells are intact after neuropathic noise. Photomicrographs of the organ of Corti from the ~32 kHz region of mice exposed to 97 dB SPL noise and evaluated either 1 month (**a**) or 16 months (**b**) after exposure. Scale bar: 20 μm (**a**-**b**).

Despite the loss of synaptic ribbons following 97 dB SPL exposure, there was no visible structural damage to the sensory epithelia of the organ of Corti, and no loss of hair cells, when inspected by light microscopy up to 16 months post exposure (Fig 5).

### Delayed Degeneration of peripheral axons and cell bodies

**Fig 6:**
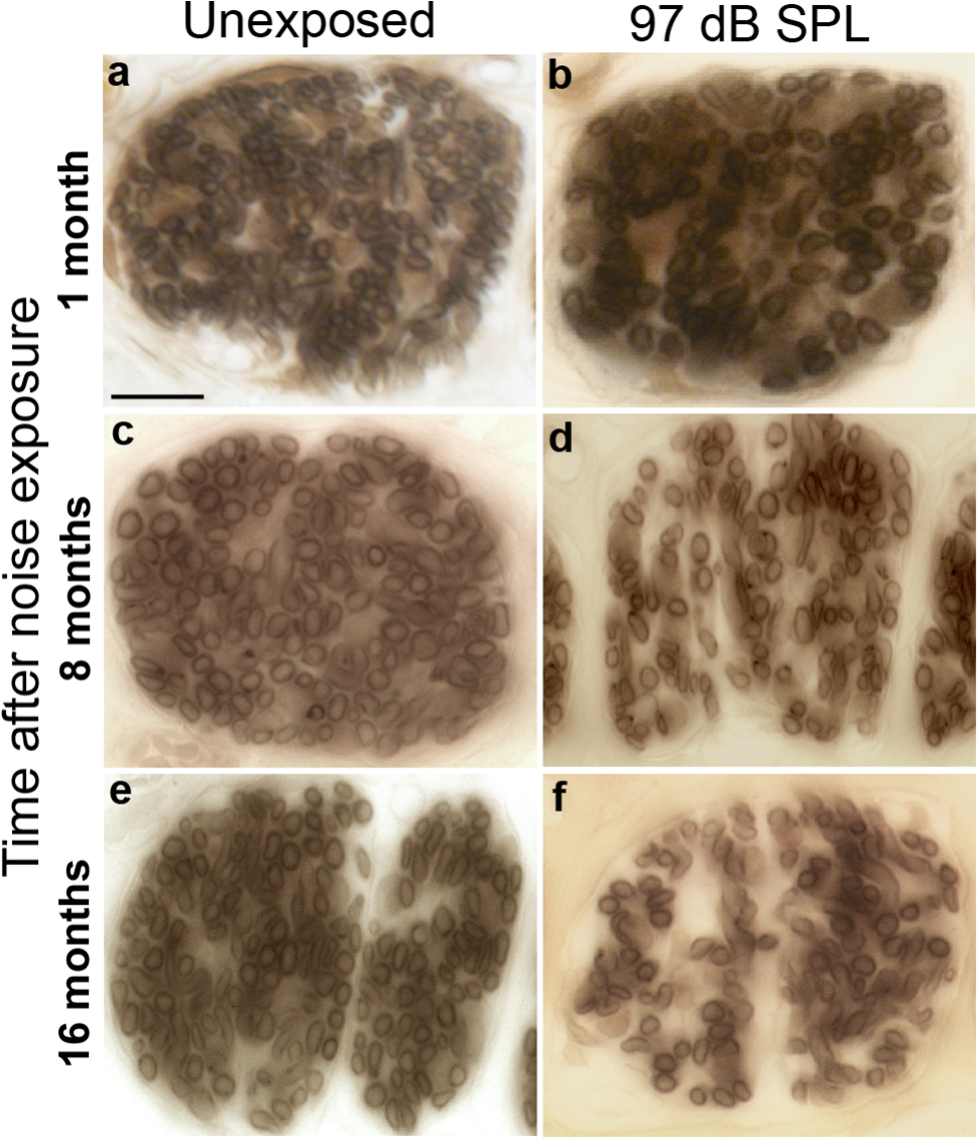
Cochlear-nerve peripheral axons can be counted in tangential sections through the osseous spiral lamina. Tangential cuts though the osseous spiral lamina in the ~32 kHz region of cochleae 1 month (**b**), 8 months (**d**) or 16 months (**f**) after exposure to neuropathic noise (97 dB SPL) demonstrate loss of peripheral axons compared to age-matched unexposed controls (**a**, **c**, **e**). These sections show fascicles of cochlear-nerve peripheral axons roughly midway between their cell bodies in the spiral ganglion and their peripheral terminals in the organ of Corti. Scale bar: 10 μm (**a**-**f**).

**Fig 7:**
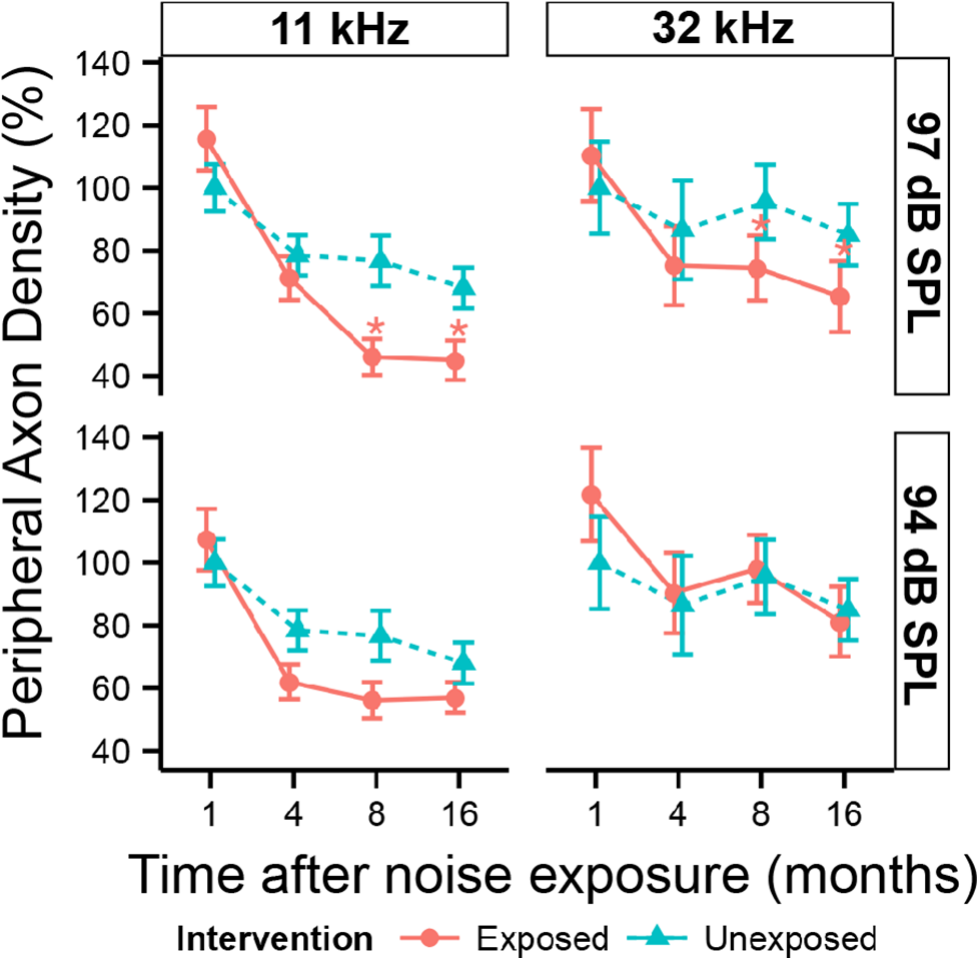
Peripheral axons of cochlear nerve fibers slowly degenerate after neuropathic noise. Density of peripheral axons as a function of post exposure time in the ~11 kHz (left) and ~32 kHz (right) region, following exposure to neuropathic (97 dB SPL) or non-neuropathic (94 dB SPL) noise. Data are group means (± SD), expressed relative to data from the youngest unexposed ears. Statistical significance of the group differences is indicated by asterisks: *: *p < 0*.*05*, **: *p < 0*.*01*. N = 3–7 animals per time and noise exposure.

To track SGN degeneration following loss of synaptic ribbons, we inspected (Fig 6) and counted (Fig 7) their myelinated, peripheral axons in cochlear sections. Tangential cuts through the osseous spiral lamina in the upper basal region (~32 kHz) of unexposed mice (Fig 6A, 6C and 6E) and mice exposed to neuropathic noise (Fig 6B, 6D and 6F) suggest a loss of cochlear-nerve peripheral axons in the neuropathic group, at both 8 months (Fig 6D), and 16 months post exposure (Fig 6F). Quantification of peripheral axons (Fig 7) confirmed the trends. Two-way ANOVA found highly significant (*p < 0*.*001*) time and exposure effects on peripheral axon counts in both frequency regions. Exposure to neuropathic noise caused significant loss of peripheral axons relative to controls at 8 months post exposure in both the apex (40.0% + 8.2%; *p < 0*.*05* by BH corrected Wilcoxon rank sum test) and base (22.1% + 5.2%; *p < 0*.*05*). At 16 months post exposure, axonal counts were reduced by 33.9% + 8.1% in the apex (*p < 0*.*05)* and 23.2% + 5.7% in the base (*p < 0*.*05)*. Exposure to non-neuropathic noise did not cause statistically significant changes in axonal counts compared to age-matched controls. The trend toward fewer axons in the 11 kHz region after non-neuropathic noise was not significant after correction for multiple hypothesis testing.

Irrespective of noise exposure, peripheral axonal counts in the apical region show early signs of retraction starting at 4 months post exposure, and decrease with age by 32.0% + 6.5% (*p < 0*.*01)*, and by 15% + 9.8% in the base in unexposed 17.5 month old animals (16 months “post exposure”) compared to 10 week old mice (1 month “post exposure”). However, this trend did not meet our criterion for statistical significance in the base (*p > 0*.*05)*.

**Fig 8:**
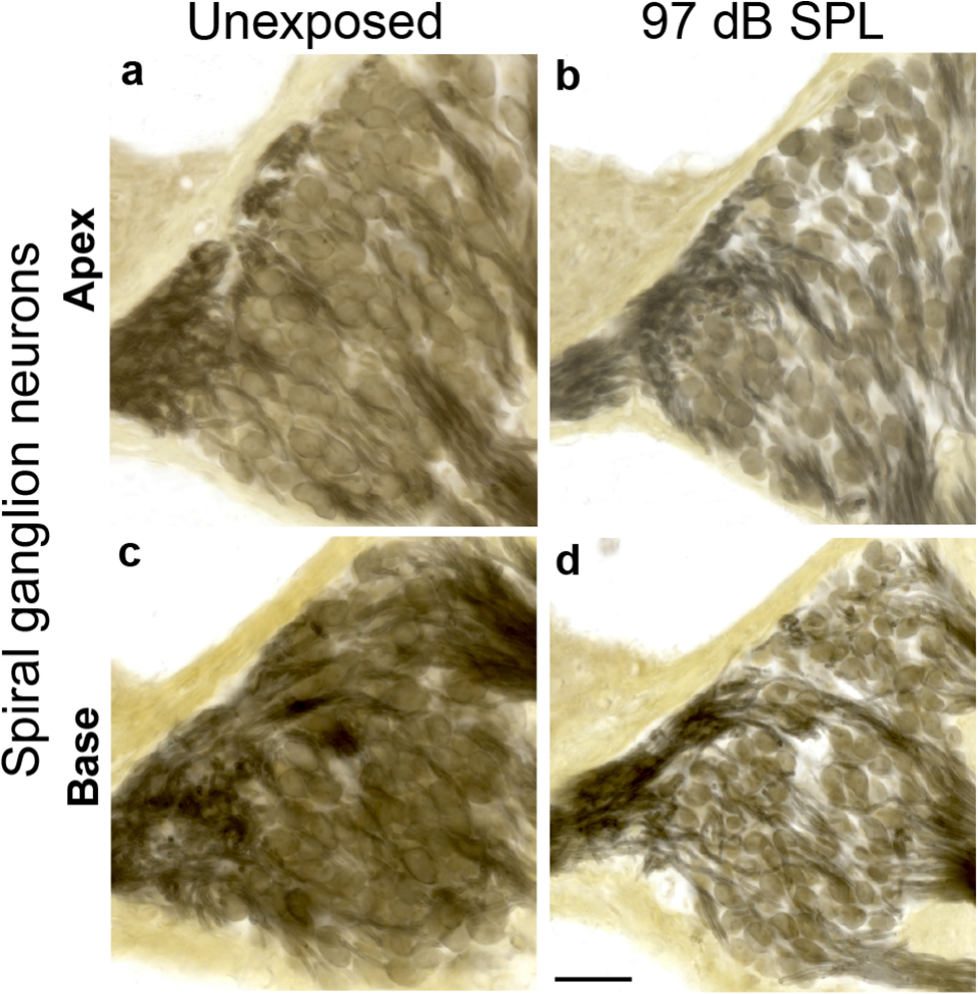
Degeneration of spiral ganglion neurons is assessed in cochlear sections after neuropathic noise. Midmodiolar sections through Rosenthal’s canal show permanent loss of spiral ganglion neurons in the apex (**b**) and base (**d**) 16 months after exposure to neuropathic noise (97 dB SPL) compared to age-matched unexposed controls (**a**, **c**). Scale bar: 50 μm (a-d).

**Fig 9:**
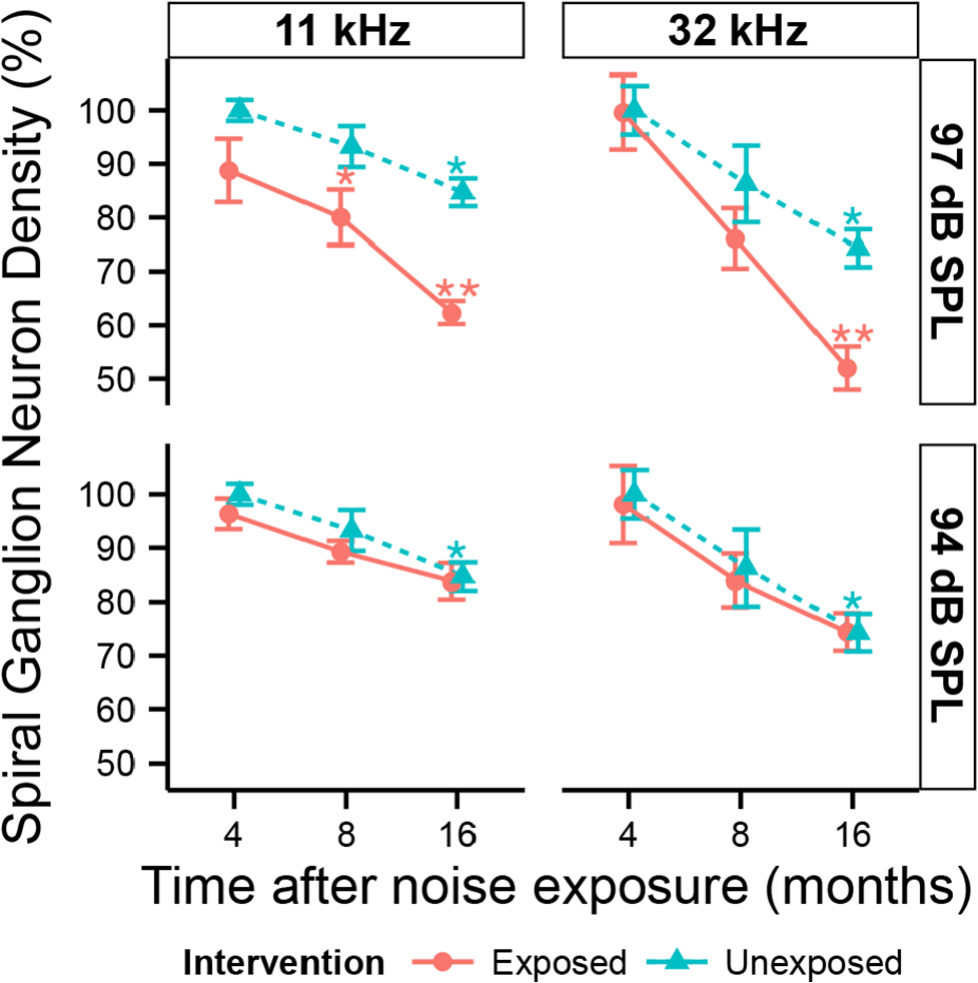
Late-onset loss of cochlear neurons following neuropathic noise. Density of spiral ganglion neuron somata as a function of post exposure time, as seen in the ~11 kHz (left) and ~32 kHz (right) regions after exposure to neuropathic (97 dB SPL) or non-neuropathic (94 dB SPL) noise. Data are group means (± SD), expressed relative to data from the youngest unexposed ears. Statistical significance of the group differences is indicated by asterisks: *, *p < 0*.*05*; **, *p < 0*.*01*. N = 3–7 animals per time and noise exposure.

With noise-induced retraction of peripheral axons of SGNs starting at 8 months post exposure, a subsequent loss of SGN somata is expected. Indeed, visual inspection of osmium-stained sections at 16 months post exposure revealed an obvious loss of SGNs in both the apex (~11 kHz region, Fig 8B) and base (~32 kHz region, Fig 8D) compared to age-matched controls (Fig 8A and 8C). Two-way ANOVA comparison of SGN counts in the base at 4, 8 and 16 months post noise exposure (Fig 9) revealed highly significant (*p < 0*.*001*) time and exposure effects. Statistically significant decreases were observed in the base at 16 months post exposure to neuropathic noise. By that time 50.0% + 4.3% (*p < 0*.*01* by BH corrected Wilcoxon rank sum test) of SGNs were missing. Interestingly, in the apex, SGN loss was significant at 8 months post exposure (14.1% + 5.7%; *p < 0*.*05*), and persisted 16 months post exposure (26.3% + 2.8%, *p < 0*.*01*). Exposure to non-neuropathic noise did not cause significant SGN loss compared to unexposed mice. Irrespective of noise exposure, SGN counts decreased with age. In the unexposed group, SGN counts at 17.5 months declined 15.3% + 2.6% (*p < 0*.*01)* in the apex and 25.7% + 3.5% (*p < 0*.*01*) in the base compared to counts at 5.5 months (4 months “post exposure”).

## Discussion

### Noise-induced cochlear synaptopathy in pubescence

The phenomenon of noise-induced primary degeneration of the auditory nerve was initially described in adult mice exposed at 16 weeks of age [2], and subsequently validated in adult guinea pigs [3]. Our study demonstrates that a similar phenomenon occurs in mice exposed at 6 weeks, i.e. just at the onset of puberty. In both the pubescent 6 week old cochlea and the young adult 16 week old cochlea, a noise exposure that produces only reversible elevation of cochlear thresholds, and no hair cell loss, nevertheless can permanently destroy 40–50% of the synapses between cochlear nerve fibers and inner hair cells in the basal regions of the cochlea.

Noise-induced threshold elevation, whether temporary or permanent, is primarily due to dysfunction, and loss of the normal contribution of outer hair cell electromotility to cochlear amplification, as evidenced by the similarity in the degree of threshold shift measured via OAEs and ABRs. If the outer hair cells recover, cochlear thresholds can recover. According to a prior study in 16 week old mice [2], thresholds for ABR recover despite substantial loss of synapses because the neuropathy is selective for fibers with high-thresholds and low spontaneous rates [35].

Noise-induced synaptopathy is thought to reflect a type of glutamate excitotoxicity [36,37]. Swelling of afferent nerve terminals is seen 48 hrs after a noise-induced TTS [6,38,39], and glutamate agonists can mimic [40,41], while glutamate antagonists can prevent this swelling [36,42]. Calcium overload is a major contributor to glutamate excitotoxicity in the central nervous system [43–45], and it is likely that the mechanisms are similar at the cochlea’s glutamatergic synapses. The enhanced sensitivity of high threshold fibers [8] may be due to the paucity of mitochondria [33,46,47], and therefore a decreased ability to buffer this calcium overload.

### The threshold for noise-induced cochlear synaptopathy

Specifying the noise-level “threshold” for cochlear neuropathy is important for several reasons. First, it defines a useful “control” group in studies aimed at deciphering molecular mechanisms. Comparing gene-expression changes at early post exposure survivals between neuropathic and non-neuropathic groups of exposed mice would likely yield a better focused list of key damage pathways than a design which compares exposed mice to unexposed controls, as has been the case for prior TTS studies [48–50].

Secondly, it is important, for possible re-design of damage risk criteria, to understand whether all TTS-producing exposures are neuropathic. Here, we showed that only a 3 dB increase in exposure level turns a non-neuropathic exposure into one that destroys up to ~50% of the synapses on cochlear sensory cells in some cochlear regions. Our finding in 6 week old mice is consistent with a prior study in 16–18 week old mice wherein a 100 dB exposure was neuropathic, whereas a 94 dB was not, despite the fact that both produced a TTS of roughly 40 dB when measured 24 hrs post exposure [51].

### Immediate vs. delayed cochlear synaptopathy

Prior work in young adult mice aged 16 weeks showed that the loss of cochlear synapses was demonstrable within 24 hrs post exposure, and neither recovered nor increased during the next 8 weeks post exposure [2]. The synaptic loss should immediately render the affected neurons non-responsive to sound, since each fiber contacts only a single IHC via a single terminal swelling [33]. Thus, after the hair cells (and thresholds) recover, there should be a good correlation between the drop in ABR suprathreshold amplitudes and the loss of cochlear synapses. Indeed, we showed a ~30% drop in ABR wave I amplitude and a ~30% drop in ribbon counts at the corresponding cochlear location at 2 weeks post exposure. The apparent recovery of synaptic counts between day one and two weeks post exposure could reflect a degree of regeneration [52], or a transient down-regulation of synaptic protein expression during and immediately after the exposure. Such a down-regulation could be part of a protective mechanism, as has been described for the post-synaptic side: cochlear nerve terminals appear to internalize glutamate receptors during a traumatic exposure to noise [53].

In noise-induced synaptopathy in the 16 week old mice, the degeneration of neuronal cell bodies in the spiral ganglion occurred at a similar pace: spiral ganglion cell loss was not detectable for several months and did not approach the magnitude of immediate synaptic loss until almost 2 years post exposure [2], but showed a reduction of ~45% at 16 mo post exposure. Here, we showed a similar delayed onset of the degeneration of cochlear nerve peripheral axons and cell bodies. At one month post exposure, there was no detectable degeneration of either peripheral axons or cell bodies, whereas by 16 months post exposure, which reflects about half the murine life expectancy, the ganglion cell loss approached the initial synaptopathy 24 hrs post exposure (~50%).

In addition to this delayed disappearance of the cell bodies and peripheral axons of cochlear neurons that have lost their peripheral synapses, we also observed delayed spatial spread of the synaptopathy itself. Although loss of synapses in the cochlear base is essentially immediate, synaptic loss also slowly spreads to the cochlear apex, where losses reach statistical significance by 16 months post exposure, when compared to age-matched controls. As reported previously [22] there is significant age-related synaptic loss even in animals maintained in the controlled acoustic environment of an animal care facility. Present results reinforce prior work suggesting that early noise exposure accelerates age-related hearing loss [12,54], as it progresses from high to low frequencies. Neuropathic changes in the apical peripheral axons could be detected earlier (4 months post exposure) than synaptic loss, at 16 months post exposure, likely because of increased sampling density for these structures at later time points.

### Hidden hearing loss: public health implications for pubescence and beyond

In general, animal experiments may not directly extrapolate to humans because human SGNs have distinctive features, such as lack of somatic myelination, and sharing of myelin sheats by multiple SGN cell bodies [55–57]. However, suprathreshold ABR may be useful in the non-invasive diagnosis of cochlear synaptopathy because noise-induced and age-related relative decrements in human ABR wave I amplitude are comparable to the relative changes we have observed in mice. Specifically, when studying human subjects with normal threshold responses, noise-exposure backgrounds explained approximately 15 to 24% of the variance in wave I amplitude [58]**.** When focusing on human subjects with normal threshold responses and tinnitus, ABR wave I amplitude was reduced on average 22% at 90 dB SPL and 24% at 100 dB SPL compared to the control subjects without tinnitus, suggesting that cochlear neuropathy contributes to tinnitus [59]. The measured increase in the ratio of ABR wave V to wave I in tinnitus subjects could be explained by 53–61% deafferentation in a computational model [59]. Subsequent studies of other subjects with tinnitus and normal auditory thresholds [59–61] have validated decreased amplitude of ABR wave 1, consistent with cochlear neuropathy. In an aging study of veterans without substantial hearing loss, the amplitude of ABR wave I decreased 38% over a 40 year span from age 30 to 70 years of age [62], again suggestive of cochlear neuropathy. Taken together, the published studies of human subjects have strongly suggested noise-induced and age-related synaptopathy that is similar in magnitude and time course (relative to the species lifespan) to the synaptopathy we have found in the current study of pubescent mice, and Kujawa and Liberman (2009) have found in young adult mice.

However, despite the strong suggestion of cochlear synaptopathy in people with otherwise normal thresholds, a definitive proof of synaptopathy is lacking because ABR measurements in people cannot yet be complemented with direct visualization and quantification of cochlear neurons and their synapses. The ongoing development of a cochlear endoscope for minimally-invasive intracochlear imaging based on two photon fluorescence may provide the missing link [63]. Specifically, we have demonstrated feasibility of cellular level imaging of control and noise-exposed murine cochlear cells—including neuronal cell bodies, nerve fibers and hair cells—without contrast dyes, through the round window, using two photon microscopy [63]. Because the autofluorescence signal appears to be generated by flavine adenine dinucleotide, which is an important cofactor in metabolic reactions, the autofluorescence signatures from the deafferented neurons may be different from those of neurons with intact afferent synapses.

In the meantime, refinements of the non-invasive electrophysiologic techniques promise to provide deeper insight into human cochlear synaptopathy. Specifically, Envelope Following Response (EFR) may be more sensitive in detecting cochlear synaptopathy than ABR because, unlike ABR, EFR reflects activity of high-threshold fibers more than low-threshold fibers (reviewed by Plack et al., 2014) [64].

Taken together, our results in an animal model suggest that a re-examination of damage risk criteria may be required to prevent cochlear synaptopathy, and the “hidden” hearing loss it produces. The early symptoms of cochlear synaptopathy, and the preferential loss of high-threshold fibers, are likely to be difficulties understanding speech in noise, when these fibers are normally activated [51]. To date, national standards for noise exposure (set by the National Institute for Occupational Safety and Health and Centers for Disease Control) are not based on age at exposure. Although exposure to high noise levels is estimated to be decreasing in the workplace [65], sales of portable listening devices are increasing, primarily among adolescents [35,66]. This has resulted in a shift of “at-risk groups” from adults to adolescents. Our data from pubescent mice contribute to the mounting evidence of cochlear neuropathy underlying “hidden” hearing loss.
